# Video education versus face-to-face education on inhaler technique for patients with well-controlled or partly-controlled asthma: A phase IV, open-label, non-inferiority, multicenter, randomized, controlled trial

**DOI:** 10.1371/journal.pone.0197358

**Published:** 2018-08-01

**Authors:** Hye Jung Park, Min Kwang Byun, Jae-Woo Kwon, Woo Kyung Kim, Dong-Ho Nahm, Myung-Goo Lee, Sang-Pyo Lee, Sook Young Lee, Ji-Hyun Lee, Yi Yeong Jeong, You Sook Cho, Jeong-Hee Choi, Byoung Whui Choi

**Affiliations:** 1 Department of Internal Medicine, Gangnam Severance Hospital, Yonsei University College of Medicine, Seoul, Korea; 2 Department of Internal Medicine, Kangwon National University Hospital, Chuncheon, Korea; 3 Department of Internal Medicine, Dongguk University Ilsan Hospital, Goyang, Korea; 4 Department of Allergy & Clinical Immunology, Ajou University Hospital, Suwon, Korea; 5 Pulmonary, Allergy & Critical Care Medicine, Hallym University Chuncheon Sacred Heart Hospital, Chuncheon, Korea; 6 Department of Internal Medicine, Gachon University Gil Hospital, Incheon, Korea; 7 Department of Internal Medicine, Seoul St. Mary’s Hospital, The Catholic University Medical College of Korea, Seoul, Korea; 8 Division of Respiratory and Critical Care Medicine, Department of Internal Medicine, CHA Bundang Medical Center, CHA University, Seongnam, Korea; 9 Division of Pulmonary and Critical Care Medicine, Department of Internal Medicine, Gyeongsang National University Hospital, Jinju, Korea; 10 Division of Allergy and Clinical Immunology, Department of Internal Medicine, Asan Medical Center, Seoul, Korea; 11 Pulmonary, Allergy & Critical Care Medicine, Hallym University Dongtan Sacred Heart Hospital, Hwaseong, Korea; 12 Department of Internal Medicine, Chung-Ang University Hospital, Seoul, Korea; Charité - Universitätsmedizin Berlin, GERMANY

## Abstract

**Background:**

Education on inhaler technique is critical for effective asthma treatment. However, traditionally used face-to-face education is time-consuming, costly, and often laborious. The current study evaluated the efficacy of a newly developed video-based inhaler technique education method.

**Methods:**

A total of 184 subjects with well-controlled or partly-controlled asthma were enrolled from 12 hospitals in South Korea from 30 November 2015 to 01 June 2016. Subjects were randomly divided into two groups in a 1:1 ratio; a control group that received face-to-face education, and a study group that received video education. All subjects received fluticasone propionate plus salmeterol xinafoate (Fluterol^®^ 250/50 inhalation capsules) for 12 weeks. The primary outcome measure was forced expiratory volume in the 1st second (FEV_1_) at 12 weeks. The secondary outcome measures were change in FEV_1_ at 4 weeks, change in asthma control test (ACT) score, and changes in various inhaler technique parameters. These measures were assessed with a non-inferiority margin of 10% between the control group and the study group.

**Results:**

FEV_1_ was significantly improved at 12 weeks in the control group and the study group. After adjustment, FEV_1_ improvement was not significantly inferior in the study group compared to the control group. The secondary outcome measures, including change in FEV_1_ at 4 weeks, ACT score, and various parameters pertaining to inhaler technique and satisfaction at 4 and 12 weeks did not differ significantly in the two groups. In subgroup analysis of elderly subjects and subjects with well-controlled asthma, FEV_1_ was significantly improved at 12 weeks in the study group but not the control group.

**Conclusion:**

The newly developed video education technique investigated functioned as a suitable substitute for face-to-face education on inhaler technique (dry powder inhalation capsule) in patients with stable asthma, particularly in elderly patients and patients with well-controlled asthma.

## Introduction

Asthma is a chronic inflammatory airway disease, the prevalence of which is increasing worldwide with changes of environment and genetic interaction [1–4]. Inhalers are the most common way to deliver asthma medications. Inhaled medications include corticosteroids, which are the most effective control drugs [5–7], and long-acting β_2_ agonists, which are used as additional therapy for poorly controlled asthma. Inhaled medication is preferred over oral medication, because inhaled medication can be delivered directly to the airways in high concentrations while minimizing systemic bioavailability and increasing the risk–benefit ratio of drugs. However, the prevalence of inhaler misuse including failure to properly prepare the inhaler, failure to correctly place the inhaler, failure to breathe in and out appropriately, failure of the patient to hold their breath after inhalation, and failure to rinse their mouth after self-administration reportedly ranges from 15–94% [8–14]. In addition, many patients evidently have a false perception of their inhaler technique [10,15]. Inhaler misuse should be corrected, because it leads to poorly controlled asthma [11,16,17]. The 2016 report published by the Global Initiative for Asthma encourages clinicians to check for inaccurate inhaler use and provide adequate education [17,18].

Various education methods of inhaler technique have been developed to improve asthma management. Written instructions on how to use an inhaler, verbal instructions augmented by physical demonstrations [19], pharmacist instructions on proper inhaler technique [20–22], physician-centered education programs [23], and teach-back methods that include patient demonstrations back to healthcare providers [24] all improve patient satisfaction, asthma control, and prognoses. Group lectures and demonstrations, videos and multimedia internet-based training [25], and teach-to-goal video programs have further improved the success rates of education on inhaler technique [26]. However, all these methods are time-consuming, costly, and labor-intensive. Some educational programs can only be implemented after intensive training of healthcare providers. Moreover, some video-based programs require specialized equipment and the installation of complex software. Because there is no widely accepted education method, many institutions use their own resources (*e*.*g*., education by a nurse or medical resident, unofficial videos) to improve the success rates of inhaler technique education.

We developed a simple new educational video that can reduce the time, cost, and effort required to educate patients on effective inhaler use. We surmised that video-based education did not need to demonstrate superiority to face-to-face education. Non-inferiority should be considered sufficient with regard to the usefulness of video-based education, because video-based education could reduce the time, cost, and effort required to educate patients on the proper use of their inhalers. We hypothesized that our newly developed education method was not inferior to the conventional face-to face education method. The current study aimed to evaluate the efficacy of this newly developed video-based education method (one-way education) with regard to with inhaler technique in terms of degree of asthma control, patient satisfaction, inhaler technique, and drug compliance in patients with well-controlled or partly-controlled asthma, compared to conventional face-to-face education by a healthcare provider (two-way education).

## Materials and methods

### Patients

This study was conducted in 12 hospitals in South Korea. Registration dates ranged from 30 November 2015 (date of first patient registration) to 01 June 2016 (date of last patient registration). Follow-up periods ranged from 22 February 2016 (end of the first follow-up) to 30 August 2016 (end of the last follow-up). Subjects were enrolled if they were adults (> 19 years), had well-controlled or partly-controlled asthma (asthma control test [ACT] score 16–25), required inhaler treatment (Fluterol^®^; Hanmi Pharmaceutical, Seoul, Korea) as determined by the investigator, and had provided written informed consent to participate in the study. Exclusion criteria were as follows: history of hypersensitivity to fluticasone propionate or salmeterol xinafoate (the components of the drug used in the study); cardiac tachycardia; untreated fungal or bacterial respiratory infection (including tuberculosis); moderate to severe bronchiectasis; status asthmaticus or asthma emergency requiring intensive treatment; chronic obstructive pulmonary disease; having received systemic steroids within 2 weeks of screening; hypersensitivity to lactose or milk; pregnant or lactating women, women who were planning to become pregnant, and women who were not willing to use appropriate methods of contraception. Patients who had received previous medication (naive, oral medication, or inhaler), except those who were currently using Fluterol^®^ or similar inhalation devices (*e*.*g*., Onbrez Breezhaler^®^ [Novartis, Basel, Switzerland], Spiriva HandiHaler^®^ [Boehringer Ingelheim, Inc., Ingelheim am Rhein, Germany]) were eligible for inclusion in this study.

### Study design

The study was designed to evaluate the efficacy and safety of video education (one-way education) compared to face-to-face education (two-way education) for asthma control in patients with well-controlled or partly-controlled asthma. It was a phase IV, open-label, non-inferiority, multicenter, randomized controlled trial. Subjects who met the inclusion criteria were randomly assigned to either the control group or the study group for 12 weeks in a 1:1 ratio, via stratified block randomization. Group assignment was not revealed to the investigators or the study subjects. The subjects were stratified by study center and prior experience with asthma inhalers. Subjects visited their registered site at weeks 4 and 12 (visits #2 and #3, respectively) to evaluate the degree of asthma control, satisfaction with inhaler use, and whether they use inhaler properly. In addition, all adverse events occurring during the study period were documented, and safety was assessed via vital signs and acute exacerbation data.

### Video education method

Subjects assigned to the study group watched a 10-minute video on a laptop computer when they visited their registered site at day 1 and weeks 4 and 12 (visits #1, #2, and #3, respectively). The video could be viewed repeatedly until the viewer could fully understand the instructions. The video was developed by Hanmi Pharmaceutical and contained detailed information on how to use the inhaler device. The video was comprised of three parts: (1) An explanation of the device’s structure by an unseen narrator, accompanied by an on-screen picture of the device; (2) device demonstration by an expert; and (3) a summary of how to use the device by an unseen narrator [https://doi.org/10.5061/dryad.7vn81m1], while showing a picture indicating the direction of the device [S1 File]. In addition, manuals on how to use inhaler medications were provided to the subjects to reinforce the knowledge acquired during the video session. The total duration of each video session was 10 minutes.

### Face-to-face education method

The face-to-face education (two-way education) method was set up by the investigators who were responsible for education on inhaler technique. The optimal education strategy for correct inhaler technique was adjusted to each subject’s needs, and inhalation errors were minimized via patient demonstration of the inhaler technique and error correction by the investigator. This process was comprised of five parts: 1) Face-to-face, step-by-step explanation of the inhaler use manual by the investigator (S1 File); 2) confirmation by the investigator that the subjects used the inhaler properly; 3) error correction and re-education according to each subject’s needs; 4) reassessment and confirmation by the investigator that the subjects used the inhaler properly; and 5) provision of a manual to the subjects to reinforce the knowledge acquired during the face-to-face education session.

### Data collection and definitions

We collected detailed data pertaining to comorbidity, including various vascular, pulmonary, neurologic, endocrine, myocardial, renal, liver, and gastrointestinal diseases. We used Charlson’s comorbidity to classify comorbidity condition. Charlson’s comorbidity index was calculated in accordance with the literature [27]. Predicted lung function (%) was calculated as previously described by Quanjer et al. [28]. This equation can be applied globally in different ethnic groups in subjects aged 3–95 years.

### Fluticasone/Salmeterol

The drug used in the inhaler system utilized by all patients in the study (Fluterol^®^) contains a combination of 250 μg fluticasone propionate and 50 μg salmeterol xinafoate per capsule. Fluterol^®^ was approved for maintenance treatment in asthma and chronic obstructive pulmonary disease by the Ministry of Food and Drug Safety of Korea on 22 January 2014. Capsules containing fluticasone/salmeterol are inserted into a small device, and to inhale the medication users must press the button and breathe through the mouthpiece rapidly.

### Study endpoints

The primary endpoint of this study was forced expiratory volume in the 1st second (FEV_1_) as determined via spirometry at 12 weeks. In stable asthma with short-term treatment, changes in FEV_1_ are more sensitive than changes in exacerbation rate and ACT score [29]. Spirometry was performed in accordance with the American Thoracic Society recommendations [30] by a specially trained nurse, The secondary outcomes assessed in each group were FEV_1_ change at 4 weeks compared to baseline, change in ACT score, change in inhaler technique score, number of critical errors, percentage of optimal inhaler technique subjects, feeling of satisfaction with inhaler (FSI-10) [31], and adherence rate (%) at 4 and 12 weeks compared to baseline (S2 File). These outcomes were observed and assessed by the investigator.

### Statistical analysis

Data obtained from the study subjects were analyzed using the full analysis set (FAS), per-protocol set (PPS), and safety set. To assess group differences in baseline characteristics, the *t*-test or Wilcoxon rank-sum test were used for continuous variables, and Pearson’s chi-square test or Fisher’s exact test were used for categorical variables. To evaluate the non-inferiority of the difference between the two groups (study group vs. control group), analysis of covariance (ANCOVA) incorporating FEV_1_ at baseline, prior experience with an inhaler for asthma, and sex was performed. If the lower limit of the two-sided 95% confidence interval of the least-square means difference between the two groups was greater than -10%, the study group was considered non-inferior compared to the control group. Post-hoc subgroup analysis was performed to assess associations between primary outcomes and age and ACT score. Power calculation analysis showed that with a change in the FEV_1_ standard deviation of 18.03% and a non-inferiority margin of 10%, a power of 90% and alpha of 0.025 would require 69 subjects per group. The confidence interval used in safety assessment was calculated based on the code developed by John C. Pezzulla (http://www.sample-size.net/confidence-interval-proportion/). All statistical tests were performed with a two-sided significance level of 0.05.

### Ethics

This study was approved by the Ethics Committee of the National Evidence-Based Healthcare Collaborating Agency. The protocol was also approved by the institutional review board of each participating center (approval dates varied, but all were between 23 November 2015 and 01 February 2016) (S3–S5 Files). All patients provided written informed consent to participation in the study. The authors confirm that all previous and ongoing trials related to this drug/intervention are registered. This current trial is registered in the ClinicalTrial.gov registry (number NCT03110874). Although registration in the ClinicalTrial.gov registry was delayed, the study complied with the CONSORT checklist.

## Results

### Study flow

**Fig 1:**
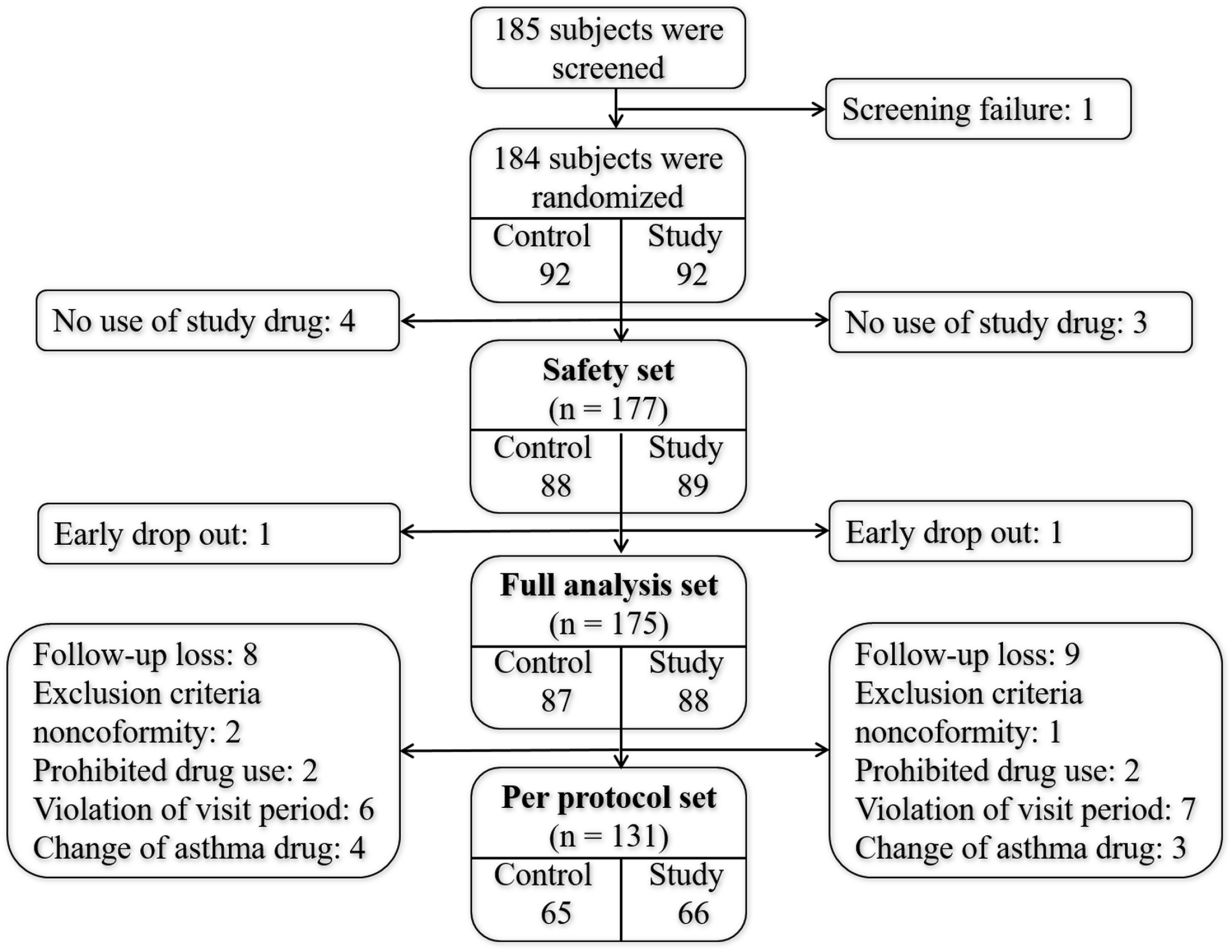
Study flow.

A total of 185 subjects were screened for eligibility. One patient who had an ACT score of 10 was excluded based on the inclusion criteria. The remaining 184 were randomized into a control group (face-to-face education/two-way education; *n* = 92) and an study group (video education/one-way education; *n* = 92). A total of 7 patients were lost to follow-up (never returned to their respective clinics after their initial visit). Thus, 177 subjects were included in the safety analysis (the safety set; control group *n* = 88, study group *n* = 89). Two early drop-outs were subsequently excluded, leaving a FAS of 175 subjects (control group *n* = 87, study group *n* = 88). Of these, 44 were excluded. Ultimately, the per-protocol set (PPS) analyzed included 131 subjects (control group *n* = 65, study group *n* = 66) (Fig 1).

### Demographics

In the control group the mean age ± standard deviation was 52.0 ± 15.3 years, and in the study group it was 52.5 ± 16.7 years (*p* = 0.62). There were significantly more male subjects in the study group (55.2%) than in the control group (37.5%) (*p* = 0.02). There were no significant differences in the distributions of height, weight, prevalence of current smokers, education level, family history of asthma, family history of allergy, or duration of asthma between the two groups. The respective proportions of subjects with prior experience using an asthma inhaler were 88.6% and 90.8% in the control and study groups (*p* = 0.64). Charlson’s comorbidity index did not differ significantly in the two groups (*p* = 0.55) (Table 1).

**Table 1:** Demographics of enrolled subjects (full analysis set).

Parameters		Control (n = 88)	Study (n = 87)	*P*-value
Age (years)		52.0 ± 15.3	52.5 ± 16.7	0.62*
**Sex (male)**		**33 (37.5%)**	**48 (55.2%)**	**0.02**†
Height (cm)		161.7 ± 8.9	163.4 ± 9.6	0.23‡
Weight (kg)		66.0 ± 13.8	65.2 ± 11.2	0.95*
Current smoker		15 (17.1%)	8 (9.2%)	0.12§
Education	Not educated	3 (3.4%)	4 (4.6%)	0.87§
	Elementary school	9 (10.2%)	13 (14.9%)	
	Middle school	12 (13.6%)	9 (10.4%)	
	High school	25 (28.4%)	27 (31.0%)	
	College	33 (37.5%)	30 (34.5%)	
	Graduate school	6 (6.8%)	4 (4.6%)	
Family history of asthma		21 (23.8%)	19 (21.8%)	0.75†
Family history of allergy		14 (15.9%)	22 (25.3%)	0.13†
Duration of asthma (years)		6.5 ± 7.5	5.7 ± 6.2	0.93*
Prior experience of inhaler for asthma		78 (88.6%)	79 (90.8%)	0.64†
Charlson’s comorbidity index		0.12 ± 0.41	0.09 ± 0.38	0.55*

*, Wilcoxon’s rank sum test;

^†^, Pearson’s chi-square test;

^‡^, Two sample t-test;

^§^, Fisher’s exact test

Significant value was presented as bold

Data were presented as mean ± standard deviation or number (%)

### FEV_1_ improvement after 12 weeks in the control and study groups

**Fig 2:**
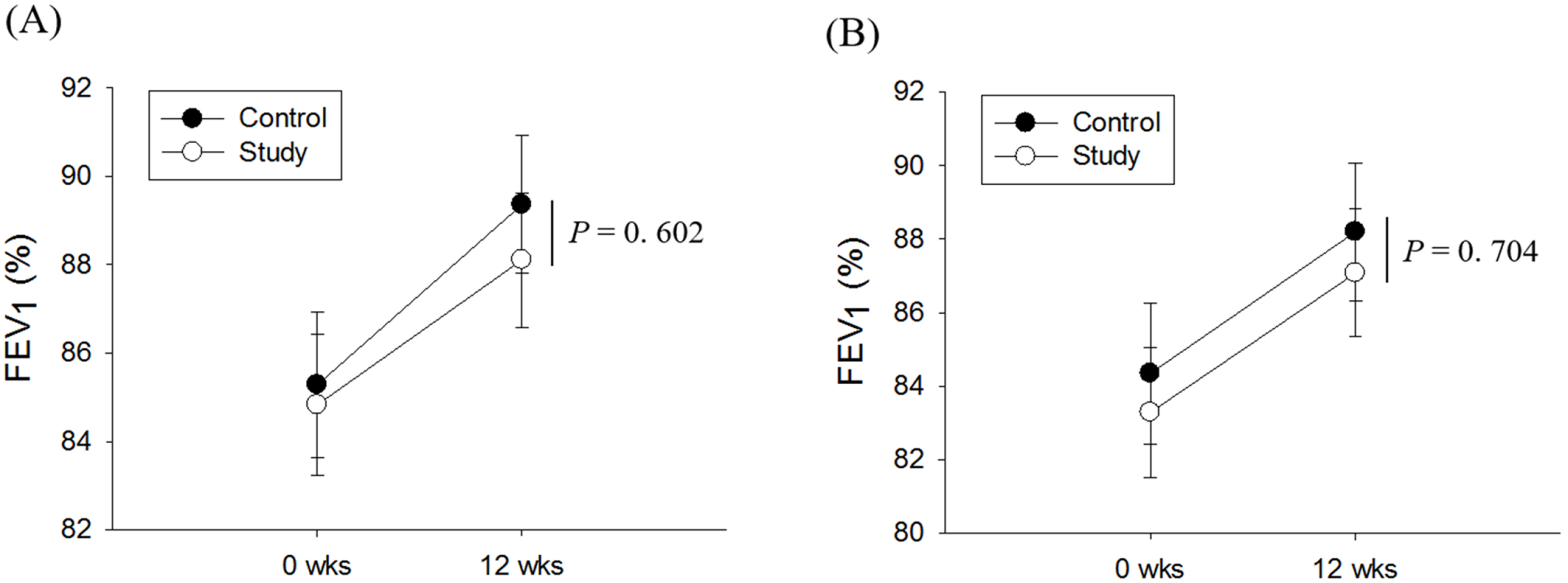
FEV_1_ improvement after 12 weeks in FAS (A) and PPS analysis (B). The *t*-test or Wilcoxon’s signed rank test were used to analyze improvement within groups, and ANCOVA was used for comparisons of improvement between the two groups. The data are presented as the mean (dot) and standard error of the mean (bar). FEV_1_, forced expiratory volume in the 1st second; FAS, full analysis set; PPS, per-protocol set.

In the FAS analysis, FEV_1_ was significantly improved in the control group (from 85.3 ± 1.7% [mean ± standard error of the mean] to 89.4 ± 1.6%; *p* < 0.01) and the study group (from 84.8 ± 1.6% to 88.1 ± 14.2%; *p* < 0.01). The difference in FEV_1_ improvement between the two groups was not significant after adjustment for FEV_1_ at baseline, prior experience using an asthma inhaler, and sex (*p* = 0.60) (Fig 2A). In PPS analysis, there was also a significant improvement in FEV_1_ in both groups, but no significant difference in the improvement between the two groups (*p* = 0.70) (Fig 2B).

### FEV_1_ improvements after 4 weeks in the control and study groups

**Fig 3:**
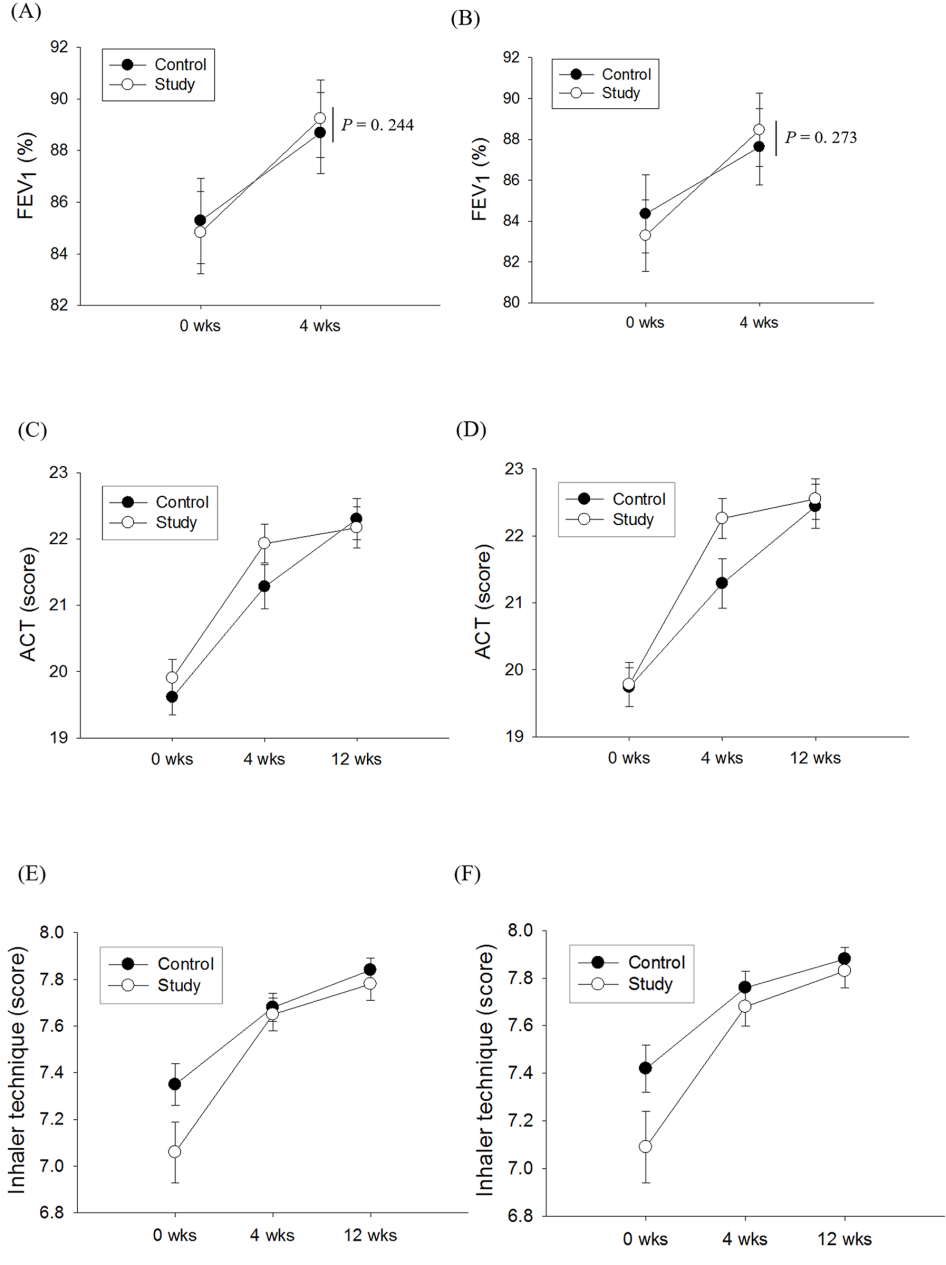
FEV_1_ improvement in the FAS (A) and PPS (B), ACT improvement in the FAS (C) and PPS (D), and inhaler technique score improvement in the FAS (E) and PPS (F). The *t*-test or Wilcoxon’s signed rank test were used to analyze improvement within groups, and ANCOVA was used for comparisons of improvement between the two groups. The data are presented as the mean (dot) and standard error of the mean (bar). FEV_1_, forced expiratory volume in the 1st second; ACT, asthma control test; FAS, full analysis set; PPS, per-protocol set.

In FAS analysis, FEV_1_ was significantly improved in the control group (from 85.3 ± 1.7% to 88.7 ± 1.6%; *p* < 0.01) and the study group (from 84.8 ± 1.6% to 89.2 ± 1.5%; *p* < 0.01). There was no significant difference between the FEV_1_ improvements in the two groups after adjustment for confounding variables (*p* = 0.24) (Fig 3A). PPS analysis showed the same trends as FAS analysis (Fig 3B).

### ACT improvements after 4 and 12 weeks in the control and study groups

In FAS analysis, in the control group there were significant improvements in ACT from baseline (19.6 ± 0.3; mean ± standard error of the mean) at 4 weeks (21.3 ± 0.3, *p* < 0.01) and 12 weeks (22.3 ± 0.3, *p* < 0.01). In the study group there were also significant improvements in ACT from baseline (19.9 ± 0.3) at 4 weeks (21.9 ± 0.3, *p* < 0.01) and 12 weeks (22.2 ± 0.3, *p* < 0.01) (Fig 3C). The results of PPS analysis were similar to those of FAS analysis (Fig 3D).

### Inhaler technique at 4 and 12 weeks in the control and study groups

In FAS analysis, in the control group there were significant improvements in inhaler technique score from baseline (7.4 ± 0.1; mean ± standard error of the mean) at 4 weeks (7.7 ± 0.1, *p* < 0.01) and 12 weeks (7.8 ± 0.1, *p* < 0.01). In the study group, there were also significant improvements in inhaler technique from baseline (7.1 ± 0.1) at 4 weeks (7.7 ± 0.1, *p* < 0.01) and 12 weeks (7.8 ± 0.1, *p* < 0.01). There were no significant differences in inhaler technique improvements between the two groups at 4 weeks (*p* = 0.86) or 12 weeks (*p* = 0.86) after adjustment for confounding variables (Fig 3E). PPS analysis yielded similar results (Fig 3F).

### Other secondary outcomes at 4 and 12 weeks in the control and study groups

In FAS and PPS analysis, the control and study groups did not differ significantly from each other with regard to the number of critical errors, the number of subjects with optimal inhaler technique, FSI-10, or adherence rates at 4 weeks or 12 weeks (Table 2).

**Table 2:** Other secondary outcomes at 4 weeks and 12 weeks according to the group.

Parameters	Set	Group	4 weeks	*P*-value*	12 weeks	*P*-value*
Number of critical error (/person)	FAS	Control	0.2 ± 0.4	0.57	0.1 ± 0.3	0.44
		Study	0.3 ± 0.5		0.2 ± 0.4	
	PPS	Control	0.2 ± 0.4	0.58	0.1 ± 0.3	0.94
		Study	± 0.4		0.1 ± 0.4	
Number of subjects with optimal inhaler technique	FAS	Control	63 (74.1%)	0.94	74 (87.1%)	0.77
		Study	64 (74.4%)		87 (86.2%)	
	PPS	Control	53 (80.3%)	0.56	59 (89.4%)	0.97
		Study	49 (75.4%)		58 (89.2%)	
FSI-10 (score)	FAS	Control	43.6 ± 5.6	0.68	44.2 ± 5.7	0.40
		Study	44.1 ± 6.2		44.9 ± 5.6	
	PPS	Control	44.0 ± 5.0	0.93	44.5 ± 4.8	0.46
		Study	44.0 ± 6.6		45.0 ± 5.5	
Adherence rate (%)	FAS	Control	92.9 ± 32.7	0.76	88.9 ± 15.3	0.94
		Study	93.6 ± 15.8		89.5 ± 15.3	
	PPS	Control	90.4 ± 16.3	0.10	90.2 ± 14.2	0.67
		Study	95.1 ± 15.3		89.5 ± 15.6	

FAS, full analysis set; PPS, per protocol set; FSI-10, feeling of satisfaction with inhaler

*P-value between two groups was obtained by ANCOVA analysis adjusted with FEV1 at baseline, prior experience of inhaler for asthma, and sex;

Data are presented as mean ± standard deviation or number (%)

### Post-hoc subgroup analysis for the primary outcome according to age and baseline ACT score

In subjects aged < 60 years and subjects with partly-controlled asthma (ACT score 16–19), FEV_1_ was significantly improved at 12 weeks in the control group and in the study group, and there was no significant difference between the two groups. However, in elderly subjects (aged ≥ 60 years) and subjects with well-controlled asthma (ACT score 20–25), FEV_1_ was significantly improved at 12 weeks in the study group but not in the control group. However, there was no significant difference between the two groups (Table 3).

**Table 3:** Subgroup analysis for primary outcome (FEV_1_, %) according to age and baseline ACT score.

Subgroup	Set	Group	Baseline	12 weeks	*P*-value*	*P*-value†
Age < 60	FAS	Control	85.6 ± 14.2	90.0 ± 12.4	< 0.01	0.23
		Study	85.5 ± 14.5	88.3 ± 13.8	< 0.01	
	PPS	Control	85.6 ± 13.7	90.2 ± 11.9	< 0.05	0.26
		Study	82.7 ± 13.6	86.8 ± 13.4	< 0.05	
Age ≥ 60	FAS	**Control**	**84.6 ± 17.9**	**88.2 ± 18.2**	**0.12**	0.80
		Study	84.0 ± 15.5	87.9 ± 14.8	< 0.01	
	PPS	**Control**	**80.7 ± 20.1**	**82.4 ± 21.5**	**0.59**	0.48
		Study	84.1 ± 15.1	87.5 ± 15.0	0.03	
ACT: 16–19	FAS	Control	83.8 ± 17.3	89.7 ± 16.7	< 0.01	0.34
		Study	85.5 ± 15.1	88.8 ± 13.6	0.01	
	PPS	Control	82.9 ± 17.3	88.8 ± 17.1	< 0.01	0.42
		Study	82.9 ± 13.3	87.3 ± 11.7	< 0.01	
ACT: 20–25	FAS	**Control**	**86.5 ± 14.0**	**89.1 ± 13.0**	**0.06**	0.97
		Study	84.4 ± 14.8	87.7 ± 14.6	< 0.01	
	PPS	**Control**	**85.3 ± 14.5**	**87.8 ± 14.0**	**0.12**	0.95
		Study	83.6 ± 15.0	86.9 ± 15.7	0.01	

ACT, asthma control test; FAS, full analysis set; PPS, per protocol set

*, *P*-value for improvement obtained by paired t-test;

^†^, *P*-value between two groups obtained by ANCOVA analysis adjusted with FEV1 at baseline, prior experience of inhaler for asthma, and sex

Insignificant *P*-value for improvement was presented as bold

Data are presented as mean ± standard deviation

### Safety

The teaching methods investigated in the current study did not directly induce adverse events. However, some adverse events that were unrelated to the teaching methods did occur. The incidence of adverse events reported in the safety set was 32.6% (95% CI 25.6–39.8%) in the control group, and it was 30.7% (95% CI 24.1–38.0%) in the study group. The most common adverse events were respiratory symptoms including upper respiratory infection, rhinorrhea, and cough (14.8% [95% CI 8.0–23.7%] and 18.0% [95% CI 12.6–30.4%] in the study group and the control group, respectively), followed by gastroenterology discomfort and muscle pain. The incidence of drug-specific adverse reactions was 4.5% (95% CI 1.2–11.1%) in the control group, and in the study group it was 5.7% (95% CI 1.9–12.8%). The incidence of serious adverse events in the control group was 1.1% (95% CI 0.0–6.2%; 1 case, road traffic accident), and in the study group it was 3.4% (95% CI 0.7–9.5%; 3 cases, neck/shoulder pain, germ cell cancer, and leg pain). There were no statistically significant differences in the rates of adverse events between the two groups (*p* > 0.05).

## Discussion

We developed a video education method that is cheap and can be easily installed on various types of hardware. The video education method was not inferior to conventional face-to-face education, which is time-consuming, costly, and labor-intensive. In order to achieve the desired therapeutic effects of an inhaler, the correct dose should be inhaled directly into the airway through the correct use of the inhaler. Incorrect inhaler use leads to insufficient drug efficacy, patient dissatisfaction, and poor asthma control and prognosis [11,16,32,33]. Therefore, proper inhaler education is crucial to achieving the desired therapeutic effects. Notably however, adequate inhalation education is not fully practiced due to a lack of medical staff, associated financial costs, and a lack of sufficient equipment. Educational videos provided by inhaler manufacturers are used unofficially in many institutions. However, quantitative comparative studies investigating inhaler education using video education or other methods are rare. In the present study, we showed that educational videos could replace face-to-face education on inhaler technique, particularly in elderly subjects and in subjects with well-controlled asthma. Clinicians could use this new method to educate their patients on correct inhaler use.

This study showed that both face-to-face education and video education promote correct inhaler use and yield similar clinical outcomes. Although many studies have reported sufficient inhaler technique success rates, studies involving comparisons of clinical outcomes between methods are rare. The endpoints in the current study included lung function (FEV_1_) and symptoms (ACT), which are the most important measures of asthma management. Improvements in these measures met minimal clinically important difference criteria [34]. In addition to correct inhaler technique performance, inhaler satisfaction and compliance associated with the video education method were not inferior to those associated with face-to-face education. In addition, video education appeared to be equally as safe and effective for providing training in the techniques required to use a new device as face-to-face education.

Interestingly, in the current study elderly subjects and subjects with well-controlled asthma benefited more from educational videos. Elderly people tend to exhibit poor device technique, and it has been suggested that poor cognition and impaired vision may be two of the underlying reasons for this [11,35,36]. Therefore, many clinicians have investigated methods to achieve proper device technique in elderly patients [37]. We found that video education was a good method for demonstrating correct inhaler technique to elderly people. This result may have been due to the slow and clear explanatory nature of the content of the educational video. In addition, patients with well-controlled asthma may be relatively poorly motivated with regard to engaging in face-to-face education. Older adults can replay the video as many times as needed, without feeling self-conscious or guilty about asking their healthcare provider for further explanation. Such phenomena may be particularly relevant in low-resource clinics like Korea where the doctor does not receive additional remuneration for the time spent providing inhaler education. Thus, an educational video could prove superior. Therefore, we recommend the use of educational videos in the elderly and in patients with well-controlled asthma.

We tried to minimize bias during the analysis, and to make the results robust. First, we adjusted critical parameters to obtain reliable results. Sex distribution differed significantly in the two groups, therefore we adjusted the analysis for sex. Although prior experience with an asthma inhaler did not differ significantly in the two groups, this factor was also adjusted for because it has been suggested that prior experience with a different inhaler device may negatively affect the results of inhaler technique education pertaining to a new device [38]. Subjects without experience will find learning the inhaler technique more difficult than subjects with experience. In addition, baseline FEV_1_ was adjusted to match baseline lung function. We adjusted the analysis for three factors using ANCOVA. Second, we conducted both FAS and PPS analysis, and the results of each were similar. Third, various parameters pertaining to inhaler technique including the number of critical errors, the number of subjects with optimal inhaler technique, FSI-10, and adherence rates were analyzed, and the results of these analyses showed the same trends.

The results of the current study are applicable to patients with well-controlled asthma and partly-controlled asthma. Patients with uncontrolled asthma should be carefully educated and managed. From the outset, we considered that patients with uncontrolled asthma would need more intensive education and management than that provided by a simple video education program. For this reason, we decided to only enroll patients with well-controlled or partly-controlled asthma.

Video education can be applied easily in most institutes. It does not require high-quality equipment, complex software, substantial space, or significant financial outlay. It is very cheap and simple. Clinicians can deliver this educational program via various types of hardware (including computers, tablets, and even mobile phones), and begin using the video immediately to educate their patients. Patients can watch such a video independently in 10 min. No help from clinicians, nurses, or paramedics is required. After watching the video, patients do not require any further help or processing.

This study had some limitations. First, it included many patients with stable asthma without recent exacerbation who had previous experience with inhaler use. These patients may not have been ideal candidates with regard to demonstrating significant improvements in symptoms and lung function. The small improvements in FEV_1_ and ACT scores in the two groups in the study may reflect this. However, if education on inhaler technique was not sufficient, clinical outcomes may have been worse than baseline. Therefore, we did not consider these to be confounding factors. Second, face-to-face education varies in different institutions and with different healthcare providers. However, we tried to reduce this variation by issuing formal instructions to all participating healthcare providers aimed at promoting standardization (at least to the extent that was possible) in this regard. Third, the asthma management regimes and the corresponding educational video investigated in this study only pertained to the use of one type of inhaler, Fluterol^®^. The effectiveness of the educational video method should be verified with various types of inhalers. Fourth, this study was not blinded and the role of the investigators in the intervention may have influenced the results because it was a multicenter intervention trial. Fifth, we excluded many women of reproductive age due to uncertainty of the safety of the use of inhalers containing long-acting bronchodilators in such patients; and this may have skewed the results. Lastly, additional long-term studies including many patients with severe asthma should be conducted, to confirm the results of the present study.

## Conclusion

The above-described newly developed educational video tool may constitute a suitable substitute for face-to-face education on inhaler technique in stable asthma patients. Beyond this, in elderly asthmatics and patients with well-controlled asthma video education may be even be more beneficial than face-to-face education. This new method has the potential to enhance patient education on inhaler technique in various contexts, and improve asthma management.

## Supporting information

S1 File Handout with usage instruction. (PDF)

S2 File Definitions of study endpoints. (DOCX)

S3 File Study protocol in Korean. (DOCX)

S4 File Study protocol in English. (PDF)

S5 File CONSORT 2010 checklist. (DOC)
