# Supplementary material for: Video education versus face-to-face education on inhaler technique for patients with well-controlled or partly-controlled asthma: A phase IV, open-label, non-inferiority, multicenter, randomized, controlled trial
Source: PLoS One. 2018 Aug 1;13(8):e0197358. doi: 10.1371/journal.pone.0197358 (PMC6070174; doi:10.1371/journal.pone.0197358)
Supplement: S1 File — (PDF) [file pone.0197358.s001.pdf]

플루테롤 한미핼러 사용법

(플루티카손프로피오네이트/살메테롤지나포산염)

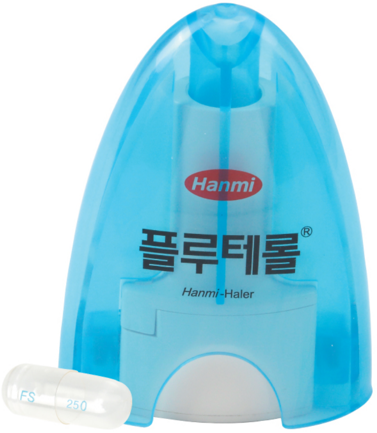

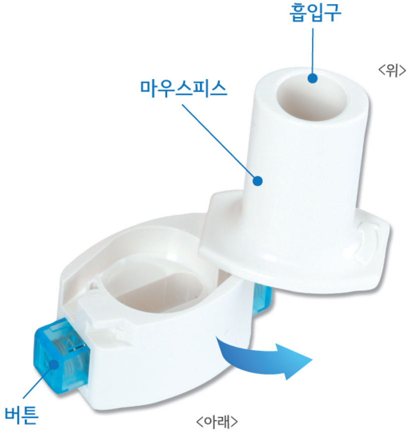

____________________ 님,　　이 약 1캡슐을 첨부된 흡입기에 장착하여 1일 2회 경구흡입 하세요.

*구강 칸디다증 또는 목이 쉬는 것을 예방하기 위해 흡입 후 양치질을 하거나 물로 입을 헹구며, 헹군 물을 삼키지 않습니다.

*한미핼러는 필요시 청소가 가능합니다. 마우스피스를 열고 따뜻한 물로 한미핼러를 헹구어, 남아있는 약물 가루를 제거하십시오. 남아있는 물을 깨끗한 수건에 털어 내고, 기구를 열린 채로 두어 공기 중에서 건조시킵니다. 반드시 다음 사용할 때에 충분히 건조된 상태에서 사용합니다.

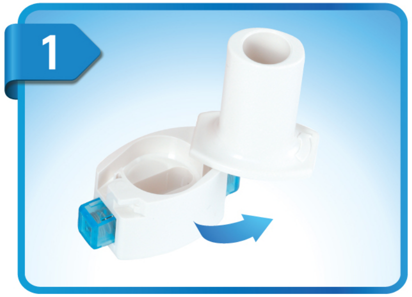

① 흡입기를 반시계방향으로 회전

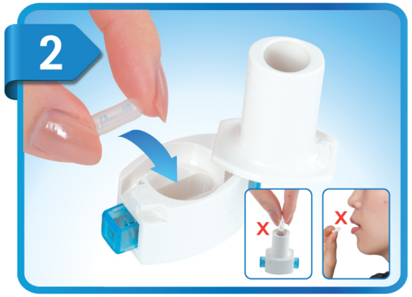

② 캡슐을 흡입기 중앙 홈에 삽입

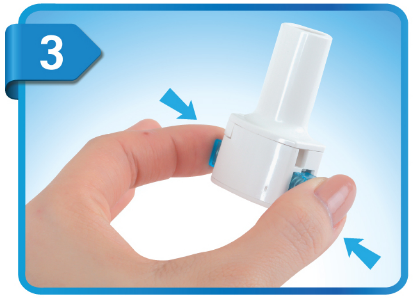

③ 양 옆의 버튼을 눌러서 캡슐을 터뜨리고 버튼을 놓음

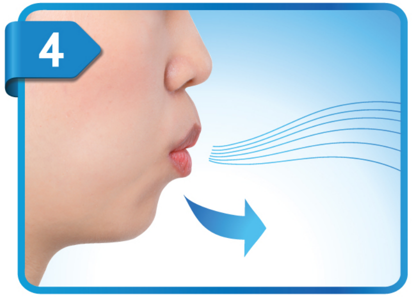

④ 흡입 전 숨을 완전히 내쉼

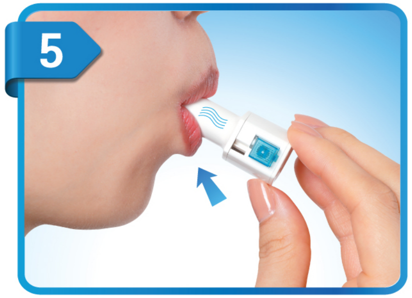

⑤ 숨을 깊게 들이마시며 약품 흡입
(캡슐이 흔들리는 소리가 들리는지 확인)

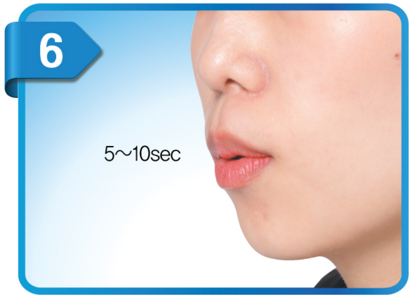

⑥ 5~10초간 숨을 참은 후 내쉼

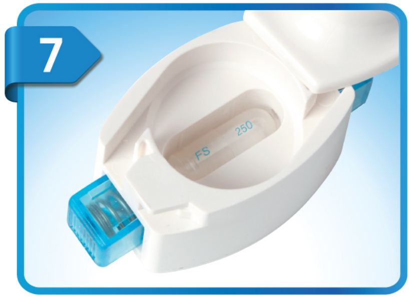

⑦ 캡슐의 분말이 비었는지 확인
(분말이 남은 경우 4번부터 다시)

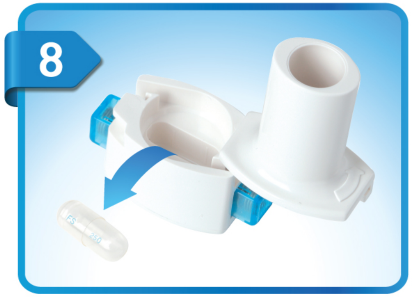

⑧ 캡슐 제거
