# Supplementary material for: Video education versus face-to-face education on inhaler technique for patients with well-controlled or partly-controlled asthma: A phase IV, open-label, non-inferiority, multicenter, randomized, controlled trial
Source: PLoS One. 2018 Aug 1;13(8):e0197358. doi: 10.1371/journal.pone.0197358 (PMC6070174; doi:10.1371/journal.pone.0197358)
Supplement: S2 File — (DOCX) [file pone.0197358.s002.docx]

1. Inhaler technique score

The process for inhaler technique was divided to 8 steps (items). One point was assigned to an item below, and the total score was checked by sum of the scores, which ranged from 0 to 8.

[Eight items for inhaler technique score]

1. Rotation of device counter-clockwise
2. Insertion of capsule into the midline hole in the device
3. Press of the buttons to break the capsule and release of the buttons – Critical item
4. Full expiration before inspiration – Critical item
5. Deep inspiration the medicine (Check the sounds of capsule) – Critical item
6. Hold of breath for 5-10 seconds and expiration
7. Check of the capsule to be empty
8. Removal of the capsule

2. Number of critical errors

The number of critical errors which are included in the above inhaler technique items (number 3-5) per person was checked.

3. Number of subjects with optimal inhaler technique

The number (or proportion) of the subjects with optimal inhaler technique who received total 8 scores in the inhaler technique checklist was assessed.

4. Feelings of satisfaction with inhaler (FSI-10)

FSI-10 includes three categories (ease or difficulty of use, portability, and usability), and compromised with 10 items. Each item was assessed from 1 score to 5 score (1, never; 2, no; 3, some yes; 4, near yes; 5, absolutely yes), and the total score was suggested.

5. Adherence rate (%)

Adherence rate was calculated by the equation below.

Adherence rate (%) = Actual used amount of medicine x 100/Planned amount of medicine
