# Supplementary material for: Video education versus face-to-face education on inhaler technique for patients with well-controlled or partly-controlled asthma: A phase IV, open-label, non-inferiority, multicenter, randomized, controlled trial
Source: PLoS One. 2018 Aug 1;13(8):e0197358. doi: 10.1371/journal.pone.0197358 (PMC6070174; doi:10.1371/journal.pone.0197358)
Supplement: S4 File — (PDF) [file pone.0197358.s004.pdf]

**Title of study**	A Multi-center, Randomized, Interventional, Open, Phase Ⅳ Clinical Trial on Education Method of Fluterol® Inhalation Capsule 250/50 μg Inhaler for Asthma Control in Patients with Partly Controlled Asthma
**Phase of study**	Phase 4
**Sponsor**	Hanmi Pharmaceutical Co., Ltd.
**Study institution and principal investigato**	Chung-Ang University Hospital (Professor Byeong-Hwi Choi) and approximately 11 other institutions in Korea
**Interventional method**	**Test group:** video based education (one-way education)<br>**Control group:** direct education (two-way education)
**Investigational product**	Fluterol® Inhalation Capsule<br>(fluticasone propionate 250 μg, salmeterol xinafoate 72.5 μg)
**Objective**	To demonstrate that video based education (one-way education) is non-inferior to face to face education as a method of education for Fluterol® Inhalation Capsule Inhaler in patients with partial controlled asthma and to assess safety..
**Study design**	A multi-center, randomized, open, non-inferiority, Phase 4 study<br><br>– Subjects who provide written informed consent to this study will be screened.<br>– Eligible subjects based on the inclusion/exclusion criteria will be randomized to either the test group (video based education, one-way education) or the control group (direct education, two-way education) and receive the investigational product for 12 weeks.<br>– The subjects will visit the institution at Weeks 4 and 12 (Visits 2 and 3) for assessment of asthma control, assessment of satisfaction for inhaler use, and education and assessment of how to use the investigational product inhaler.<br>– Adverse events (AEs) occurring during the investigational product treatment period will be collected and safety will be assessed based on vital signs and status of acute exacerbation.<br><br>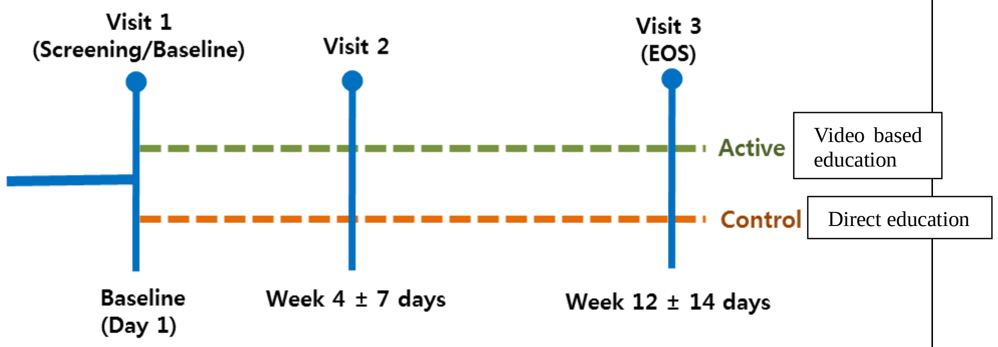<br>[Study design diagram]<br><br>A total of 2 groups<br>1) Test group: video based education (one-way education)<br>1) The subject will watch a video on the use of Fluterol® Inhalation Capsule Inhaler in an independent space (during the video based education, the subject will be allowed to repeatedly watch the video until he/she thinks that he/she has sufficiently understood how to use the inhaler and the time taken for the video based education will be measured.)<br>2) The investigator will check the subject performing each stage*.<br>3) The inhaler user’s manual will be dispensed to the subject.<br>2) Control group: direct education (two-way education)

	1) The investigator will give a demonstration of each stage using Fluterol® Inhalation Capsule inhaler technique checklist*.<br>2) The investigator will check the subject performing each stage*.<br>3) If the subject uses the inhaler in an incorrect manner, the investigator will correct it.<br>4) The investigator will re-check the subject performing each stage* (the time taken for the direct education will be measured).<br>5) The inhaler user's manual will be dispensed to the subject.<br><br>* Placebo will be used when the investigator gives a demonstration of how to use the inhaler and checks how the subject uses the inhaler.
**Indication**	Partially controlled asthma
**Sample size**	96 subjects per group, approximately a total of 192 subjects
**Inclusion/exclusion criteria**	**Inclusion criteria**<br>Subjects must meet all of the following criteria.<br>1. Adult at the age of 19 or older.<br>2. Patient with partially controlled asthma (Asthma Control Test score 16~24)<br>3. Written informed consent to study participation.<br><br>**Exclusion criteria**<br>Subjects who meet any of the followings cannot participate in the study.<br>1. History of hypersensitivity reactions to the investigational product (Fluterol®) or any of its component.<br>2. Cardiac tachyarrhythmia.<br>3. Untreated respiratory fungal, bacterial, or tuberculous infection.<br>4. Moderate to severe bronchiectasis<br>5. Planned use of the investigational product (Fluterol®) as primary treatment for patients with status asthmaticus, patients with an asthmatic crisis*, or patients with chronic obstructive pulmonary disease requiring intensive treatment.<br>* Asthmatic crisis: acute exacerbation of asthma meeting at least one of the followings.<br>• Asthma related emergency room visit or hospitalization within 2 weeks prior to screening.<br>• Administration of systemic steroids within 2 weeks prior to screening.<br>• Inhalation of rescue medication at least 10 times/day for the treatment of acute exacerbation of asthmatic symptoms within 2 weeks prior to screening.<br>6. Administration of systemic steroids within 2 weeks prior to screening.<br>7. Hypersensitivity reactions to lactose and milk.<br>8. Pregnant and lactating women or women who are planning to be pregnant or who are unwilling to use appropriate methods of contraception during the study.<br>9. Previous use of the investigational product (Fluterol®) or a similar inhaler (Onbrez, Spiriva) at least once.<br>10. Participation in another clinical study for drugs or medical devices to receive the investigational product or undergo a procedure using the investigational device within 4 weeks prior to participation in this study.<br>11. Individual considered by the he investigator to be inappropriate for study participation due to other reasons.
**Method of administration**	The investigational product will be inhaled for 12 weeks, twice daily (1 capsule per administration).
**Duration of study**	Approximately 20 months from the IRB approval (this may be changed depending on the subject enrollment rate).
**Assessment items**	**Efficacy endpoints**

and methods	1. Primary endpoint<br>Percent change in FEV1 (% prediction) at Week 12 from baseline in each group.<br><br>2. Secondary endpoints<br>1) Percent change in FEV1 (% prediction) at Week 4 from baseline in each group.<br>2) Change in asthma control (ACT) at Weeks 4 and 12 from baseline in each group.<br>3) Change in inhaler technique score at Weeks 4 and 12 from baseline in each group<br>4) Number of critical errors at Weeks 4 and 12 in each group.<br>5) Proportion of subjects with optimal inhaler technique at Weeks 4 and 12 in each group.<br>6) Satisfaction for the inhalation drug at Weeks 4 and 12 in each group (FSI-10 score).<br>7) Adherence rate (%) at Weeks 4 and 12 in each group.<br><br>**Safety endpoints**<br>AEs, vital signs, incidence rate of acute exacerbation.
**Statistical analysis**	**1. Primary efficacy endpoint**<br>- Percent change in FEV1 (% prediction) at Week 12 from baseline in each group.<br>For FEV1 (% prediction), descriptive statistics (number of subjects, mean ± standard deviation (SD), median, range (minimum, maximum)) will be presented for baseline and Week 12 values and percent change at Week 12 from baseline ((Week 12 value – baseline value) X100/baseline value) in each group. To test the non-inferiority between the two groups (test group – control group) in terms of the percent change, analysis of covariance (ANCOVA) will be performed with baseline FEV1 (% prediction) and 'use of asthma treatment' as covariates and two-sided 95% confidence intervals (CIs) will be presented. The non-inferiority of the test group to the control group will be demonstrated if the lower limit of the two-sided 95% CI is > -10.<br><br>**2. Secondary efficacy endpoints**<br>1) Percent change in FEV1 (% prediction) at Week 4 from baseline in each group<br>For FEV1 (% prediction), descriptive statistics (number of subjects, mean ± SD, median, range (minimum, maximum)) will be presented for baseline and Week 4 values and percent change at Week 4 from baseline ((Week 4 value – baseline value) X100/baseline value) in each group and the inter-group comparison for the percent change will be analyzed using ANCOVA with baseline FEV1 (% prediction) and 'use of asthma treatment' as covariates.<br><br>2) Change in asthma control (ACT) at Weeks 4 and 12 from baseline in each group<br>For asthma control test (ACT), descriptive statistics (number of subjects, mean ± SD, median, range (minimum, maximum)) will be presented for baseline, Weeks 4 and 12 values, and change at Weeks 4 and 12 from baseline in each group and the inter-group comparison for the difference between the baseline and each time point (Weeks 4 and 12) will be analyzed using ANCOVA with baseline ACT and 'use of asthma treatment' as covariates.<br><br>3) Change in inhaler technique score at Weeks 4 and 12 from baseline in each group<br>For the total score, for which 1 point is assigned to an item checked O and 0 point to an item checked X on the inhaler technique checklist, descriptive statistics (number of subjects, mean ± SD, median, range (minimum, maximum)) will be presented for baseline, Weeks 4 and 12 values, and change at Weeks 4 and 12 from baseline in each group and the inter-group comparison for the difference between baseline and each time point (Weeks 4 and 12) will be analyzed using ANCOVA with baseline total score and 'use of asthma

	treatment' as covariates.<br><br>4) Number of critical errors at Weeks 4 and 12 in each group<br>For number of critical errors at Weeks 4 and 12, descriptive statistics (number of subjects, mean ± SD, median, range (minimum, maximum)) will be presented in each group and the inter-group comparison at Weeks 4 and 12 will be analyzed using ANCOVA with 'use of asthma treatment' as a covariate.<br><br>5) Proportion of subjects with optimal inhaler technique at Weeks 4 and 12 in each group<br>For subjects with optimal inhaler technique at Weeks 4 and 12 in each group, frequency and percentage will be presented in each group and the inter-group comparison will be analyzed using Cochran-Mantel-Haenzel test with 'use of asthma treatment' as a covariate.<br><br>6) Satisfaction for the inhalation drug at Weeks 4 and 12 in each group (FSI-10 score)<br>For subject satisfaction (FSI-10 score), descriptive statistics (number of subjects, mean, SD, median, minimum, maximum) will be presented for Weeks 4 and 12 results in each group and the inter-group comparison will be analyzed using ANCOVA with 'use of asthma treatment' as a covariate.<br><br>7) Adherence rate (%) at Weeks 4 and 12 in each group<br>For adherence rate (%) at Weeks 4 and 12 Visits, descriptive statistics (number of subjects, mean ± SD, median, range (minimum, maximum)) will be presented in each group and the inter-group comparison for mean difference in adherence rate (%) at Weeks 4 and 12 Visits will be analyzed using ANCOVA with 'use of asthma treatment' as a covariate<br><br>**3. Safety endpoints**<br>1) AEs<br>Treatment-emergent AEs (TEAEs) will be used to for summary and analysis of AEs (TEAEs are AEs that did not exist before dosing and occur after dosing or AEs that did exist before dosing but worsen after dosing).<br>For TEAEs, adverse drug reactions, and serious AEs, frequency and percentage will be presented in each group and the inter-group homogeneity will be compared using Pearson's chi-square test or Fisher's exact test.<br><br>2) Vital signs<br>For vital signs, intra-group and inter-group comparisons will be carried out.<br>- Intra-group comparison: For continuous variables, descriptive statistics will be presented for baseline and end-of-study values and the difference between baseline and end-of-study values and mean changes will be analyzed using paired t-test or Wilcoxon's signed rank test. For categorical variables, contingency tables will be prepared and analyzed using McNemar's test.<br>- Inter-group comparison: For continuous variables, the difference between baseline and end-of-study values will be comparatively analyzed using two-sample *t*-test or Wilcoxon's rank sum test. For categorical variables, the inter-group homogeneity will be analyzed using Pearson's chi-square test or Fisher's exact test.<br><br>3) Incidence rate of acute exacerbation<br>For acute exacerbation, frequency and percentage will be presented and the inter-group homogeneity will be comparatively analyzed using Pearson's chi-square test or Fisher's exact test.
